# Supplementary material for: Efficacy of Integrase Strand Transfer Inhibitors and the Capsid Inhibitor Lenacapavir against HIV-2, and Exploring the Effect of Raltegravir on the Activity of SARS-CoV-2
Source: Viruses. 2024 Oct 13;16(10):1607. doi: 10.3390/v16101607 (PMC11512360; doi:10.3390/v16101607)
Supplement: Supplementary file 1 [file viruses-16-01607-s001.zip › viruses-3219444-supplementary.pdf]

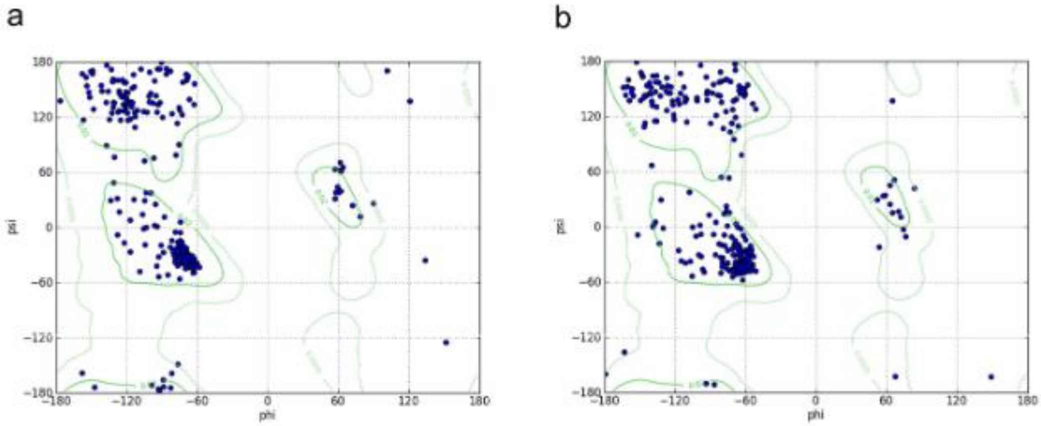


Supplementary Figure S1: Ramachandran plot of (a) SIV integrase, monomer A (PDB ID: 6RWM) compared to (b) HIV-2 integrase monomer (minimized AlphaFold model). Comparison was carried out with the UCSF Chimera software [46]

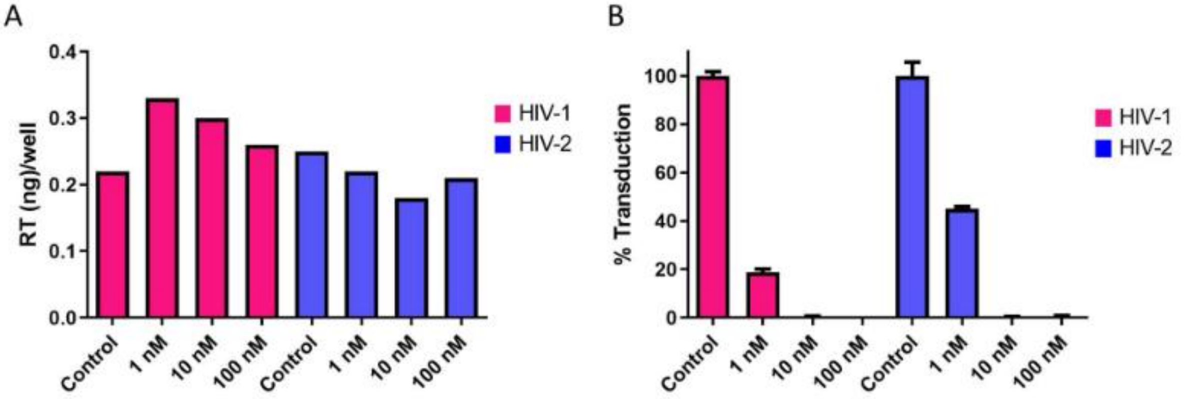


Supplementary Figure S2: A) Reverse Transcriptase (RT) activity of pseudovirions produced in the presence of 1, 10, and 100 nM of lenacapavir. B) Transduction efficiency of pseudovirions produced in the presence of 1, 10, and 100 nM of lenacapavir. Transduction experiments were performed in triplicates.

Supplementary Table S1: Sourced data from literature on the effectiveness of the inhibitors against HIV-2

| **Reference** | **Assay type** | **Raltegravir** | **Elvitegravir** | **Dolutegravir** | **Bictegravir** | **Cabotegravir** | **Lenacapavir** |
|---|---|---|---|---|---|---|---|
| Roquebert et al., 2008 [25] | In vitro phenotypic studies | 2.4 nM | 0.7 nM | - | - | - | - |
| Zheng et al.,2014 [24] | In vitro phenotypic | - | 0.3 - 0.9 nM | - | - | - | - |
| Smith et al., 2015 [20] | In vitro phenotypic | - | - | 1.1nM | - | - | - |
| Smith et al. 2018 [22] | Culture-based, single-cycle | - | - | - | - | 1.2 - 1.7nM | - |
| Smith et al. 2019 [21] | Culture-based, single-cycle | - | - | - | 1.4 to 5.6 nM | - | - |
| Link et al., 2020 [23] | In vitro clinical isolates | - | - | - | - | - | $EC_{50}$ value of 885 pM |
